# The Use and Functionality of Electronic Prescribing Systems in English Acute NHS Trusts: A Cross-Sectional Survey

**DOI:** 10.1371/journal.pone.0080378

**Published:** 2013-11-20

**Authors:** Zamzam Ahmed, Monsey Chan McLeod, Nick Barber, Ann Jacklin, Bryony Dean Franklin

**Affiliations:** 1 The Centre for Medication Safety and Service Quality, UCL School of Pharmacy, London, United Kingdom; 2 Pharmacy Department, Imperial College Healthcare NHS Trust, London, United Kingdom; University Hospitals of Geneva, Switzerland

## Abstract

**Objectives:**

To describe current use of electronic prescribing (EP) in English acute NHS hospital trusts, and the use of multiple EP systems within the same hospital.

**Design:**

Descriptive cross-sectional postal survey.

**Setting:**

Acute NHS hospital trusts in England.

**Participants:**

The survey was sent to chief pharmacists in all acute English NHS hospital trusts in 2011. Where trusts comprised multiple hospitals, respondents were asked to complete the questionnaire for their main acute hospital.

**Main Outcome Measures:**

Prevalence of EP use in acute NHS hospitals; number of different EP systems in each hospital; stages of the patient pathway in which EP used; extent of deployment across the hospital; comprehensiveness regarding the drugs prescribed; decision support functionalities used.

**Results:**

We received responses from 101 trusts (61%). Seventy (69%) respondent hospitals had at least one form of EP in use. More than half (39;56%) of hospitals with EP had more than one system in use, representing 60 different systems. The most common were systems used only for discharge prescribing, used in 48 (48% of respondent hospitals). Specialist chemotherapy EP systems were second most common (34; 34%). Sixteen specialist inpatient systems were used across 15 hospitals, most commonly in adult critical care. Only 13 (13%) respondents used inpatient electronic prescribing across all adult medical and surgical wards. Overall, 24 (40%) systems were developed ‘in-house’. Decision support functionality varied widely.

**Conclusions:**

It is UK government policy to encourage the adoption of EP in hospitals. Our work shows that EP is prevalent in English hospitals, although often in limited clinical areas and for limited types of prescribing. The diversity of systems in use, often within the same hospital, may create challenges for staff training and patient safety.

## Introduction

Recent studies report prescribing errors in 8.9 to 14.7% of inpatient and discharge medications in English hospitals [1]–[3]. Electronic prescribing (EP) is widely advocated as a potential solution to improve patient safety as well as efficiency [4]–[6]. In the UK, EP is widespread in primary care [7], but less prevalent in secondary care [8]. The National Programme for IT (NPfIT), led by England's Connecting for Health, was set up in 2002 with the goal of introducing a single electronic care record connecting all general practices and hospitals in England, including hospital EP. Full implementation was expected by 2010, but system deployment lagged behind this timescale [9], [10]. In September 2011, the UK government announced the dismantling of NPfIT; NHS hospital trusts are now making their own choices in procuring technologies such as EP.

Literature quantifying and describing the extent of EP adoption in UK secondary care is scarce, yet vital for effective planning. An informal survey conducted thirteen years ago suggested that while only one in ten hospitals had some form of EP at that time, most had plans to introduce EP in the future [11]. A survey of UK National EP Forum attendees in 2010 revealed that 82% of 56 NHS trusts were either ‘thinking of implementing’ or ‘currently implementing’ EP [12]. A more recent paper reports on experiences of EP implementation, based on a survey of EP conference attendees representing 55 (33%) of English NHS hospital trusts [13]. However, these were convenience samples and unlikely to be generalisable. Previous studies have also described EP as either being “used” or “not used” [11]–[13], in spite of systems varying widely in terms of the stages of the patient pathway in which they are used, extent of deployment across the organisation, comprehensiveness with respect to the drugs that can be prescribed, and the extent of decision support used. More than one system may also be used in the same hospital, with potential patient safety implications. These issues have not yet been explored.

Our aim was to describe the use of EP in English acute NHS hospitals. We specifically describe the stages of the patient pathway in which each system was used, its extent of deployment, comprehensiveness with respect to drugs prescribed, the decision support functionalities used, and the use of multiple EP systems within the same hospital.

## Methods

### Ethics Statement

Ethics approval was obtained from the UCL School of Pharmacy ethics committee; the local NHS Research Ethics Committee confirmed that NHS ethics approval was not required. Consent was implied if respondents returned the questionnaire.

### Study design and data collection

We conducted a cross-sectional descriptive census of acute NHS trusts in England, using a self-completed postal questionnaire. Questions relating to EP formed part of a larger questionnaire which also explored other aspects of hospital medication systems; only the aspects relating to EP are presented here. Questions were based on our experience of studying EP implementation in England [8] plus previous work in this field [11]; the questionnaire was developed according to established good practice [14]. Initial pilot work included testing several iterations of questions with a range of health care professionals. Later versions were piloted with 15 hospital pharmacists of varying experience across four trusts; two researchers each observed respondents as they completed the questionnaire to identify any problems during completion, in addition to requesting feedback. The final questionnaire included questions on trust demographics, and twelve questions about EP (appendix S1). We included specific questions exploring the extent to which systems could be used to prescribe warfarin, continuous intravenous infusions, insulin, and drugs which require a tapering dose, as these are reported to be challenging to prescribe electronically [13]. We asked respondents to include any form of EP operational in at least one ward or clinical area.

Our target respondents were trust chief pharmacists, who were encouraged to delegate questionnaire completion to colleagues as appropriate. Respondents were requested to complete the questionnaire for their main acute hospital if their trust comprised multiple hospitals. A list of all acute NHS trusts in England was obtained from NHS Choices [15], giving 165 eligible trusts at the time of the study. We used the following methods to potentially increase our response rate [16]: (i) a pre-notification letter posted to chief pharmacists in June 2011; (ii) questionnaire sent with a covering letter and a postage paid return envelope in July 2011; (iii) a follow up reminder letter posted to all non-responders four weeks later, and (iv) an electronic reminder sent to non-responders for whom we had email addresses in October 2011. The covering letter and questionnaire stated that all responses would remain confidential and that data would be anonymised. However, respondents were asked to provide their name and contact details if they were willing to be contacted for further clarification if required.

### Data Analysis

We used Excel 2007 for data entry and descriptive analysis, and Minitab 16.2.2 to compare key features of respondent and non-respondent organisations. Data entry for a random sample of 20% of returned questionnaires was checked by a second researcher. Systems used solely for clinical decision support for dosing (but not prescribing) specific drugs, such as oral anti-coagulants, were excluded from analysis. EP systems were subdivided based on the stage(s) of the patient pathway in which they were used (inpatient, discharge or outpatient), and their characteristics described. We considered a system used in all adult medical and surgical wards to be hospital-wide (or in the case of paediatric hospitals, all paediatric medical and surgical wards); this was because even hospitals with extensive use of EP may have one or more clinical areas, such as critical care or the emergency department, where EP is not used. We performed analyses by hospital, and by unique system-hospital pair (USHP). The latter was defined as one EP system implemented in one hospital; the same commercial EP system in two different hospitals was counted as two USHPs, as systems may be used differently in different settings. Any unclear responses were reviewed by a second researcher and a joint decision made as to interpretation. Where necessary, respondents were contacted to request further information. Where respondents did not state the number of wards in the relevant hospital or reported bed numbers instead, the required information was obtained from the trust's website. Information on commercial systems was checked against supplier websites and a database of NHS information technology (accessed 30 January 2012) [17] as the same system was sometimes referred to by different names.

## Results

### Respondents

We received responses from 101 trusts (61%). Two respondents completed questionnaires on behalf of all the hospitals within a trust: one for five hospitals and one for two hospitals. These were analysed with the other responses, all of which were based on the main acute hospital as requested. There were no statistically significant differences between respondent and non-respondent trusts in numbers of acute hospitals, number of wards at the main acute site, or types of service provided (table 1). A total of 25 respondents were contacted to clarify answers or request further information.

**Table 1:** Characteristics of responding versus non-responding trusts.

Characteristics	Respondents (n = 101 trusts)	Non-respondents* (n = 64 trusts)	Statistical analysis
Median number of acute hospitals in trust (range)	1 (1 – 5)	1 (1– 5)	p = 0.08; Mann-Whitney test
Median number of wards at main acute hospital (range)	25 (3– 65)	23 (1– 44)	p = 0.12; Mann-Whitney test
Services provided by main acute hospital	Adults (n = 13) or paediatrics (n = 1) only: 14 (14%) vs Mixed: 87 (86%)	Adults (n = 2) or paediatrics (n = 3) only: 5 (8%) vs Mixed: 59 (92%)	p = 0.35; chi square test with Yates correction

* Data obtained from the trust websites.

### Prevalence of EP use

**Figure 1:**
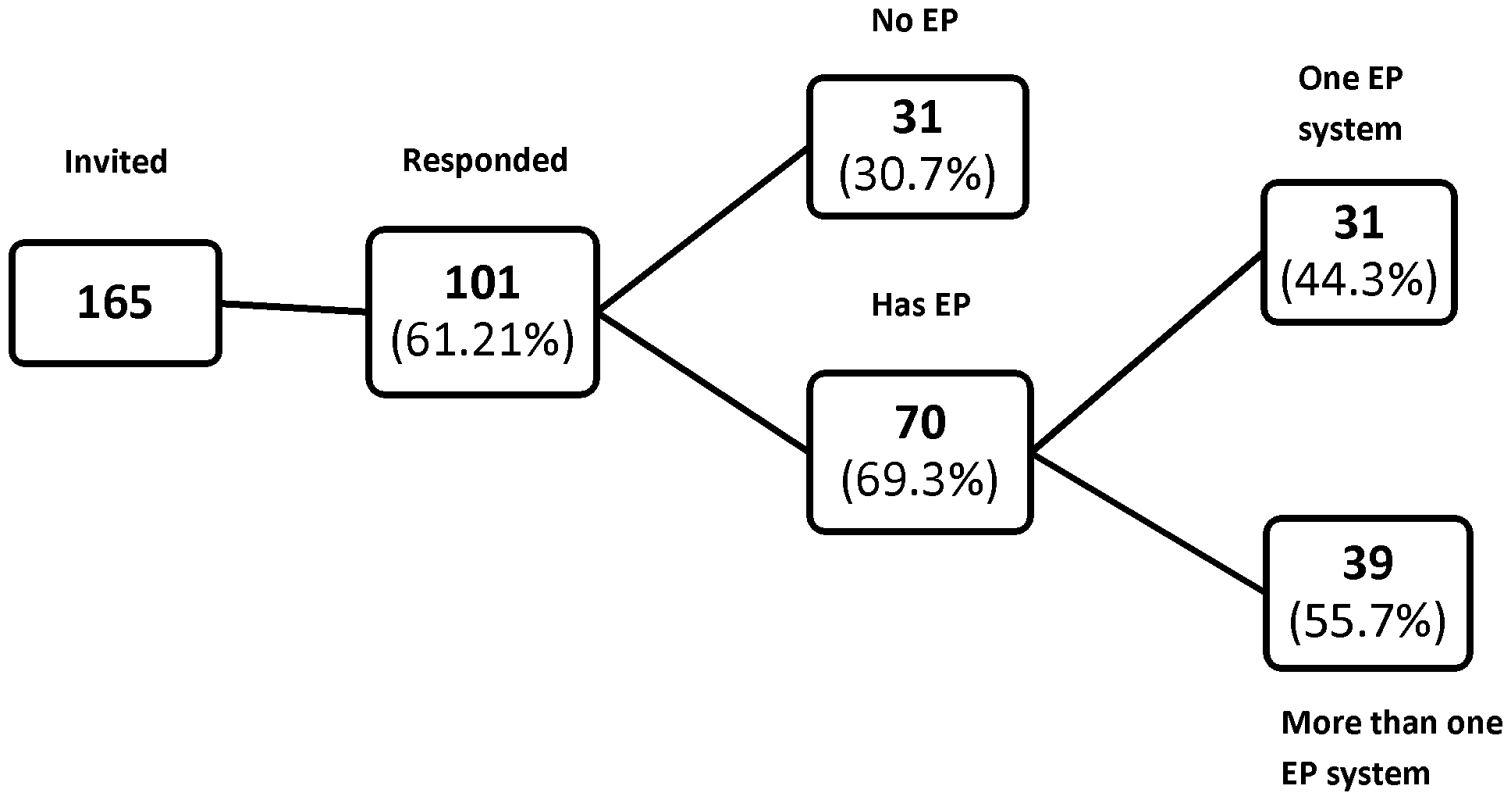
Summary of electronic prescribing (EP) use among respondents. Numbers in brackets refer to percentages of the total in the previous box.

More than two thirds (70; 69%) of respondent hospitals had at least one form of EP in use at the time of our survey, with more than half of these having more than one system (39; 56%). Twenty seven had two EP systems, eight had three systems and four had more than three (figure 1).

### Stages of the patient pathway and extent of organisational deployment

Table 2 summarises the types of EP system reported. The most common were systems used only for discharge prescribing, reported by almost half (48;48%) of all respondent hospitals. In most cases these were specialist discharge prescribing systems, but in some hospitals, commercially available systems that could also be used for inpatient prescribing were being used solely for discharge. Some hospitals had multiple discharge systems used in different clinical areas. Specialist chemotherapy EP systems were the second most common, used in 34 (34%) of respondent hospitals; two hospitals each had two different chemotherapy systems in operation. General inpatient prescribing was less common. Only 13 (13%) of respondents reported hospital-wide inpatient prescribing; all were also used for discharge. In addition, sixteen specialist inpatient systems were used across 15 respondent hospitals, most commonly in adult critical care. Excluding chemotherapy systems, 30 (30%) of respondent hospitals had some form of inpatient EP. Outpatient EP was rare. Only one hospital used EP for inpatient, discharge and outpatient prescribing; this hospital had a system developed in-house which was used in all clinical areas.

**Table 2:** Number of respondent hospitals using electronic prescribing (EP) at different stages of the patient pathway and with different levels of organisational deployment.

Type of prescribing	Number of hospitals (% of 101 respondents)	Comments
*Generalist inpatient prescribing systems*		
Generalist inpatient prescribing system in all adult medical and surgical wards (+/− other clinical areas)	13 (13%)	All 13 also used for discharge prescribing; one also used in outpatients; four also used in adult critical care
Generalist inpatient prescribing system in some clinical areas	3 (3%)	All 3 also used for discharge prescribing in these clinical areas
*Specialist inpatient prescribing systems*		
Adult critical care	11 (11%)	None used for discharge
Paediatric critical care	1 (1%)	
Neonatal care	1 (1%)	
Renal	3 (3%)	
*Specialist chemotherapy prescribing systems*		
Prescribing of chemotherapy only	34 (34%)	36 systems used across 34 hospitals; 12 used for inpatients and at discharge; 17 used in inpatients alone; three used at discharge alone; four used only for daycase chemotherapy
*Discharge prescribing*		
Standalone discharge prescribing system	48 (48%)	55 systems used across 48 hospitals; 40 used on all adult medical and surgical wards; 15 used on specific ward(s) only
*Outpatient prescribing*		
Standalone outpatient prescribing system	2 (2%)	One hospital-wide outpatient system; one system used in the emergency department only

Each EP system could be used in more than one stage of the patient pathway (e.g, inpatient and discharge), and some hospitals had more than one system. Numbers therefore do not add to 100%.

### The systems used

**Figure 2:**
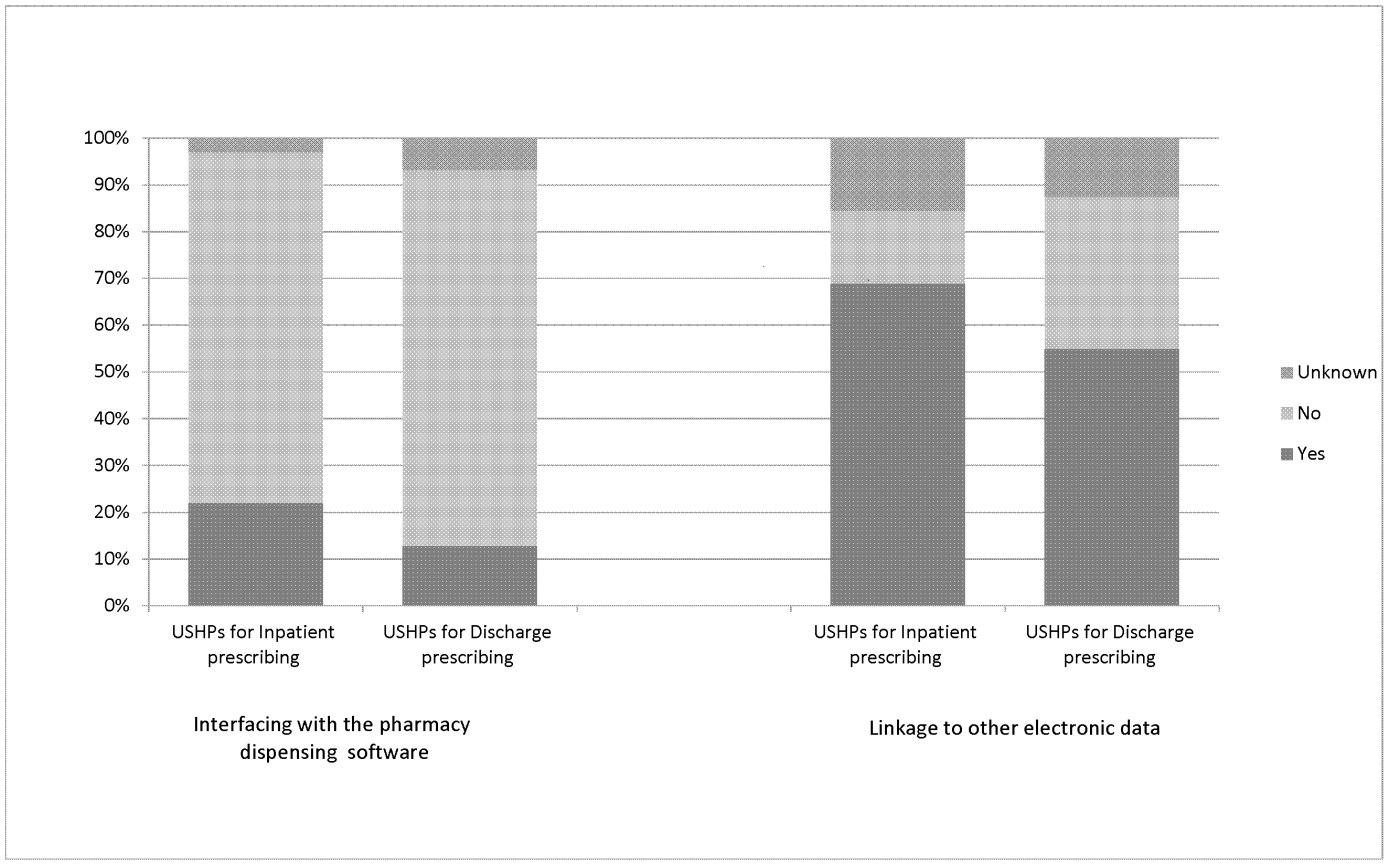
Linkage of unique system-hospital pairs (USHPs) used for inpatient (n = 32) and discharge (n = 71) prescribing with pharmacy dispensing systems and other electronic systems such as patient administration systems or clinical test results. ‘Unknown’ comprises responses for ‘not sure’, and missing data. Systems used solely for chemotherapy are excluded.

A total of 60 different systems were operational across respondent hospitals. There were 125 USHPs. Twenty four systems were developed ‘in-house’, representing 40% of systems and 19% of USHPs. Three of these were reported to be the product of joint collaboration between the relevant trust and a commercial vendor. The remainder were commercial EP systems. Two specialist cancer care systems were the most commonly used (ChemoCare and Aria), followed by a commercially available discharge system (Sunquest ICE) and another commercially available system (JAC) which can be used for inpatient, discharge and/or outpatient prescribing, followed by a specialist system used for critical care (Metavision). In some cases the same commercial system was used differently in different hospitals. For example, one such system was used hospital-wide for discharge prescribing in two hospitals, and for both inpatient and discharge prescribing on specific wards in another five. Figure 2 shows the extent to which systems were interfaced with the pharmacy dispensing software and other electronic systems such as the patient administration system or clinical test results. Interfaces with pharmacy systems were less common than interfaces with other systems, with systems used for discharge less likely to be interfaced than those used for inpatient prescribing.

### Decision support functionalities

**Figure 3:**
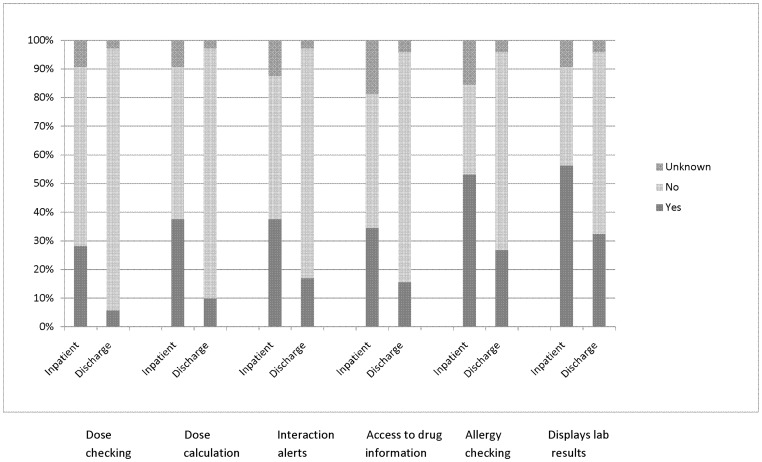
Key patient-safety related decision support functions for unique system-hospital pairs used for inpatient (n = 32) and discharge (n = 71) prescribing. ‘Unknown’ comprises responses for ‘not sure’, ‘not applicable’, and missing data. Systems used solely for chemotherapy are excluded.

There was wide variation in the decision support functionalities in use. Drug name selection from a menu was common (102; 82% of all 125 USHPs); most of these systems (71; 70%) also allowed free text prescribing. In ten and six cases respectively, respondents were not sure or selected “not applicable”. Figure 3 shows the key safety-related decision support features used in the systems for inpatient and discharge prescribing; those used for discharge generally had less decision support functionality. Excluding chemotherapy systems, half of the 32 USHPs used for inpatient prescribing allowed different levels of prescribing authority for different groups of prescribers (n = 16) and eleven (34%) could be used to order laboratory tests. Eleven (34%) and eighteen (56%) respectively did not support these functionalities while for the remainder, respondents were unsure. Drug stock level checking was a rare feature; its use was reported for only 15% (n = 5) of these USHPs, it was not used for 78% (n = 25) and in two cases respondents were unsure.

### Comprehensiveness with respect to drugs prescribed

Excluding systems used solely for chemotherapy, of the remaining 32 inpatient USHPs, 20 (63%, with 2 further respondents unsure) allowed users to prescribe continuous intravenous infusions, 17 (53%; 5 unsure) supported prescribing of tapering doses and 22 (69%; 4 unsure) supported warfarin prescribing. Sliding scale insulin seemed to be the most challenging to prescribe electronically (11; 34%, plus five unsure and two selecting ‘not applicable’). Supplementary paper-based prescribing was also reported for drugs such as heparin, gentamicin, vancomycin, controlled drugs and medication administered via syringe driver. Of the 13 hospitals using inpatient EP in all adult medical and surgical wards, all but one (8%) reported the need for supplementary paper prescription charts.

## Discussion

Some form of electronic prescribing is widely utilised in English secondary care. However, only one respondent hospital had a hospital-wide system used for inpatient, discharge and outpatient prescribing. A more common model is the use of specialised EP systems for chemotherapy prescribing, and/or in specific clinical areas, and/or for discharge prescribing alone. Use of EP for discharge prescribing is common and generally hospital-wide. Multiple systems often co-exist within the same hospital.

A strength of our study is that we were able to apply a census approach; we included all acute NHS trusts in England to document a picture of current practice that was as complete as possible. In contrast to previous work in this field [11]–[13], we captured the uptake and functionalities of all EP systems in respondent hospitals, exposing for the first time the extent of multiple EP systems within a single hospital. We have also described the stages of the patient pathway in which system were used, their extent of deployment, comprehensiveness with respect to drugs prescribed, and decision support functionalities used. Weaknesses are that our response rate, at 61%, was slightly lower than the 65% generally regarded as acceptable [18]. However, this is similar or higher than similar surveys in the USA and UK (response rates of 28%, 40%, 51%, 63% [12], [19], [20], [21]. We think it unlikely that trusts without EP were less likely to respond as the EP questions formed only one part of a wider survey of medication systems which was applicable to all English hospitals. Our survey was addressed to chief pharmacists as they were likely to have a broad overview of the systems in use together with an understanding of key clinical features; it is possible that other potential respondents such as the organisation's information technology team may have responded differently. We did not formally assess reliability or validity of our questionnaire; however questions were factual in nature and our one-to-one piloting suggested the questionnaire had high face and content validity. We did not ask specific questions about outpatient or day case EP systems; the data in table 2 reporting prevalence of EP in these areas may therefore be an under-estimate. There are also other aspects of the systems used which we did not explore, such as audit reporting functions and record keeping between successive admissions. Finally, we captured data on only the main acute hospital within multi-site trusts, which could have underestimated the number of systems in such trusts.

Our findings suggest that EP is more widespread than previously reported in the UK [11]–[13]. International comparisons are difficult as there are few similar studies. A recent US study reports 34% of hospitals as having computerised prescriber order entry for medication in 2011 [19], similar to our figure of 31% for inpatient EP. However, it is unclear if the US figure includes use in some clinical areas, as we do, or refers only to hospital-wide implementation. An earlier US survey presented similar findings to ours, reporting hospital-wide computerised prescriber order entry in 17% of hospitals with a further 11% of hospitals using it on at least one unit in 2008 [21]. However a different survey tool was used and it is not clear to what extent the findings are directly comparable. A similar proportion of English and US inpatient systems interface with the hospital pharmacy dispensing system (22% UK; 22% US) [20].

Our study reveals a wide range of EP systems used across England, with many hospitals running several systems concurrently, and with the same systems used differently in different organisations. While hospital-wide inpatient EP was uncommon, the use of EP for discharge prescriptions was prevalent, probably due to the discharge prescribing process being less complex than for inpatients. However, discharge systems were generally more basic in decision support. The high prevalence of EP being used specifically for cancer care is likely to have been driven by regional funding supporting cancer care provision in England. The wide variation in systems and how they are used is likely to create challenges for health care professionals who may have to use multiple systems within a given organisation, and will almost certainly need to learn how to use different systems if they move between organisations. The patient safety consequences of this diversity are not yet known, but there are potential risks associated with different systems having different decision support features for example. While concerns have been raised about variation in inpatient paper drug charts, resulting in calls for a national drug chart for England [22], the much wider diversity in electronic prescribing, as reported here, has not previously been highlighted. Of additional concern was that many inpatient systems did not facilitate the prescribing of high risk drugs such as sliding scale insulin and warfarin, leading to concomitant paper systems. A patient's medication records may therefore be split between electronic and paper media, with risks of medication prescribed on paper being overlooked. A recent report [6] for the Minister of Health in England suggests implementation of EP should be a priority for hospitals' IT development. While we support this stance, it is important to recognise that most of the literature demonstrating the benefits of inpatient EP has studied single hospital-wide systems, mostly in the USA. Our work suggests that in the near to mid-term future, prescribing in English hospitals will be often be delivered by a melange of multiple electronic and paper systems. This presents substantial challenges to the design of systems interfaces, training of the mobile international workforce, and the design of safe systems of working, if EP is to deliver its expected benefits.

Unanswered questions include the patient safety implications of having multiple EP systems within the same hospital, and of running parallel electronic and paper systems. It is also not clear how best to manage this diversity, nor whether this is a problem in other countries. Future research should focus on these issues.

## Conclusions

EP is prevalent in English hospitals, although often in limited clinical areas and for limited types of prescribing. The diversity of systems in use will create challenges for interfacing between systems, staff training, and patient safety.

## Supporting Information

Appendix S1 Copy of questionnaire.
(DOCX)
