# Supplementary material for: The Use and Functionality of Electronic Prescribing Systems in English Acute NHS Trusts: A Cross-Sectional Survey
Source: PLoS One. 2013 Nov 20;8(11):e80378. doi: 10.1371/journal.pone.0080378 (PMC3835329; doi:10.1371/journal.pone.0080378)
Supplement: Appendix S1 — Copy of questionnaire. (DOCX) [file pone.0080378.s001.docx]

**APPENDIX S1 : Copy of questionnaire**


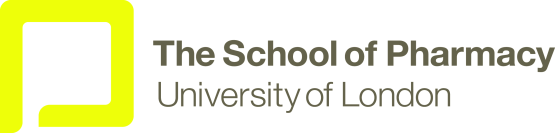


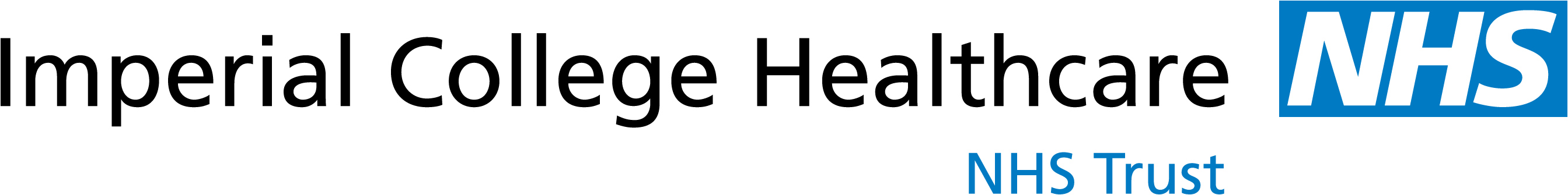


**Centre for Medication Safety and Service Quality**

**National survey of medication systems in English NHS hospitals**

Thank you for taking part in this survey.

This survey aims to identify which in-patient and discharge medication systems are

currently in use across the NHS.

Your response is invaluable to us as it will contribute to the knowledge and understanding of medication systems used in the NHS, and inform future development of strategies to:

**(1) reduce medication errors, (2) streamline hospital medication systems and (3) reduce wasted medications.**

As a thank you for your participation, we will send you a copy of the results once the national survey is complete.

**Please answer the questions in relation to the main acute hospital in your trust.**

**If your trust has multiple acute hospitals, please choose one of these on which to base the questionnaire.**

Only one questionnaire is required for each trust.

We appreciate that you might not be familiar with all the systems used in your hospital. Please complete the questionnaire as fully as you can and feel free to ask colleagues as appropriate. There is also a ‘not sure’ option for some questions.

Your answers will remain confidential.

The questionnaire will take approximately 20 to 30 minutes to complete.

Please return your completed questionnaire using the freepost envelope provided

by **Friday 22^nd^ July 2011**

Thank you for your time, we really appreciate it.

If you have any questions about this survey please feel free to contact us:

Monsey McLeod

[monsey.mcleod@imperial.nhs.uk](mailto:monsey.mcleod@imperial.nhs.uk)

TEL: 020 3313 0521

FAX: 020 3311 1342

Zamzam Ahmed

[zamzam.ahmed@phd.pharmacy.ac.uk](mailto:zamzam.ahmed@phd.pharmacy.ac.uk)

TEL: 020 7874 1272

FAX: 020 7387 5693

**PART ONE**

This section is about the medication processes and resources currently in use at your hospital.

**A: About your hospital**

| 1. **What is the name of the trust that you work in?** | ____________________________________________ |
| --- | --- |
| 1. **How many acute hospitals are there in this trust?** | __________acute hospitals |
| 1. **What is the name of the hospital that you are answering this questionnaire for?** *Please answer the questions in relation to the main acute hospital in your trust. If your trust has multiple acute hospitals, please choose one of these on which to base the questionnaire.* | ____________________________________________ |
| 1. **What in-patient group(s) does this hospital treat?** | Adults only  Paediatrics only  Mixed adult and paediatrics |
| 1. **Approximately how many in-patient wards are there in this hospital?** | __________in-patient wards |

**C: (Questions about medication supply and storage on inpatient wards)**

**B: (Questions about pharmacy service)**

**D: (Questions about medication administration, policies and guidance)**

**PART TWO**

This section is about current and/or planned electronic prescribing systems at your hospital.

**E: Electronic prescribing systems**

| Thinking about **all in-patient and discharge** services in your hospital, please answer the following questions about electronic prescribing. **In this part, please also include any systems used on intensive care, maternity and mental health wards.**  *Examples of electronic prescribing systems are:*   - *Comprehensive hospital wide prescribing systems (e.g. JAC, Cerner)* - *Speciality targeted applications/software (e.g. ChemoCare, Varian)* - *Systems relating to a specific part of the prescribing process (Electronic discharge prescribing)* | | | | | | |
| --- | --- | --- | --- | --- | --- | --- |
| 1. **Does your hospital have any electronic prescribing system in use at the moment?** | | | | | Yes | No |
|  |  | | | | (continue to Question 21) | (skip to Question 30) |
|  | | | | | | |
| 1. **Is there more than one electronic prescribing system in use in your hospital at the moment?** | | | | | | |
|  | | Yes  No  Not sure |  | If Yes, please insert number of systems: __________________ | | |

| 1. **Please insert the name of the electronic prescribing system(s) you have in the hospital.**   *Examples of electronic prescribing systems are:*   - *Comprehensive hospital wide prescribing systems (e.g. JAC, Cerner)* - *Speciality targeted applications/software (e.g. ChemoCare, Varian)* - *Systems relating to a specific part of the prescribing process (Electronic discharge prescribing)* | | | | |
| --- | --- | --- | --- | --- |
|  | **System 1**  **____________** | **System 2**  **____________** | **System 3**  **____________** | **System 4**  **____________** |

| 1. **How long has the system(s) been in place?** | | **System 1** | **System 2** | **System 3** | **System 4** |
| --- | --- | --- | --- | --- | --- |
| a. | **<1 year** |  |  |  |  |
| b. | **≥ 1 < 2 year** |  |  |  |  |
| c. | **2 – 5 year** |  |  |  |  |
| d. | **> 5 year** |  |  |  |  |
| e. | **Not sure** |  |  |  |  |
|  | Please add any other comments you have about the questions on this page. | | | | |

| **24. Please answer all statements from (a to c) for each system you have in place.** | | | | | |
| --- | --- | --- | --- | --- | --- |
| **The system you have allows:** | | **System 1** | **System 2** | **System 3** | **System 4** |
| a. | **Prescribing for in-patients** | Yes  No  Not sure | Yes  No  Not sure | Yes  No  Not sure | Yes  No  Not sure |
| b. | **Prescribing for discharge** | Yes  No  Not sure | Yes  No  Not sure | Yes  No  Not sure | Yes  No  Not sure |
| c. | **Others** , **please specify** | __________________ | __________________ | __________________ | __________________ |
|  | Please add any other comments you have. Insert the letter of the point you are referring to i.e. a. (comment) and name of system if necessary. | | | | |

| **25. Please answer all statements from (a to h) for each system you have in place.** | | | | | |
| --- | --- | --- | --- | --- | --- |
| **The system you have is in routine use in:** | | **System 1** | **System 2** | **System 3** | **System 4** |
| a. | **Adult intensive therapy units** | Not applicable  All wards  Some wards  One ward  No wards  Not sure | Not applicable  All wards  Some wards  One ward  No wards  Not sure | Not applicable  All wards  Some wards  One ward  No wards  Not sure | Not applicable  All wards  Some wards  One ward  No wards  Not sure |
| b. | **Paediatric intensive therapy units** | Not applicable  All wards  Some wards  One ward  No wards  Not sure | Not applicable  All wards  Some wards  One ward  No wards  Not sure | Not applicable  All wards  Some wards  One ward  No wards  Not sure | Not applicable  All wards  Some wards  One ward  No wards  Not sure |
| c. | **Adult medical wards** | Not applicable  All wards  Some wards  One ward  No wards  Not sure | Not applicable  All wards  Some wards  One ward  No wards  Not sure | Not applicable  All wards  Some wards  One ward  No wards  Not sure | Not applicable  All wards  Some wards  One ward  No wards  Not sure |
| d. | **Adult surgical wards** | Not applicable  All wards  Some wards  One ward  No wards  Not sure | Not applicable  All wards  Some wards  One ward  No wards  Not sure | Not applicable  All wards  Some wards  One ward  No wards  Not sure | Not applicable  All wards  Some wards  One ward  No wards  Not sure |
| e. | **Paediatric medical wards** | Not applicable  All wards  Some wards  One ward  No wards  Not sure | Not applicable  All wards  Some wards  One ward  No wards  Not sure | Not applicable  All wards  Some wards  One ward  No wards  Not sure | Not applicable  All wards  Some wards  One ward  No wards  Not sure |

| **25. Continued. Please answer all statements from (a to h) for each system you have in place.** | | | | | |
| --- | --- | --- | --- | --- | --- |
| **The system you have is in routine use in:** | | **System 1** | **System 2** | **System 3** | **System 4** |
| f. | **Paediatric surgical wards** | Not applicable  All wards  Some wards  One ward  No wards  Not sure | Not applicable  All wards  Some wards  One ward  No wards  Not sure | Not applicable  All wards  Some wards  One ward  No wards  Not sure | Not applicable  All wards  Some wards  One ward  No wards  Not sure |
| g. | **Cancer services** | Not applicable  All wards  Some wards  One ward  No wards  Not sure | Not applicable  All wards  Some wards  One ward  No wards  Not sure | Not applicable  All wards  Some wards  One ward  No wards  Not sure | Not applicable  All wards  Some wards  One ward  No wards  Not sure |
| h. | **Others, please specify type of wards** | __________________ | __________________ | __________________ | __________________ |
|  | Please add any other comments you have. Insert the letter of the point you are referring to i.e. a. (comment) and name of system if necessary. | | | | |

| **26. Which best describes your system for each statement? Please answer all statements from (a to f) for each system you have in place.** | | | | | | | | | |
| --- | --- | --- | --- | --- | --- | --- | --- | --- | --- |
| **The system is:** | | | **System 1** | | **System 2** | | **System 3** | | **System 4** |
| a. | **An in-house designed system** ‘originally designed internally within the Trust’ | | Yes  No  Not sure | | Yes  No  Not sure | | Yes  No  Not sure | | Yes  No  Not sure |
| b. | **Supplied by an external software supplier** | | Yes  No  Not sure | | Yes  No  Not sure | | Yes  No  Not sure | | Yes  No  Not sure |
| c. | **A stand alone application**  **‘**operates without other programs’ | | Yes  No  Not sure | | Yes  No  Not sure | | Yes  No  Not sure | | Yes  No  Not sure |
| d. | **Is linked with, or includes, the pharmacy dispensing software** | | Yes  No  Not sure | | Yes  No  Not sure | | Yes  No  Not sure | | Yes  No  Not sure |
| e. | **Is linked to other** **systems/ software in the hospital** *e.g. laboratory reports* | | Yes  No  Not sure | | Yes  No  Not sure | | Yes  No  Not sure | | Yes  No  Not sure |
| f. | **Is interfaced with other technologies** *e.g. bar-coding, electronic pumps.* | | Yes  No  Not sure | | Yes  No  Not sure | | Yes  No  Not sure | | Yes  No  Not sure |
|  | Please add any other comments you have. Insert the letter of the point you are referring to i.e. a. (comment) and name of system if necessary. | | | | | | | | |
| **27. Which best describes your system for each statement? Please answer all statements from (a to o) for each system you have in place.** | | | | | | | | | |
| **The system currently offers:** | | **System 1** | | **System 2** | | **System 3** | | **System 4** | |
| a. | **Dose checking**  ‘checks that dose is within normal dose range’ | Not applicable  Yes  No  Not sure | | Not applicable  Yes  No  Not sure | | Not applicable  Yes  No  Not sure | | Not applicable  Yes  No  Not sure | |
| b. | **Dose calculations**  *e.g. calculates dose per weight, calculate infusion rate, etc.* | Not applicable  Yes  No  Not sure | | Not applicable  Yes  No  Not sure | | Not applicable  Yes  No  Not sure | | Not applicable  Yes  No  Not sure | |
| c. | **Free text prescribing option**  ‘i.e. typing drug name without selecting from a list of drugs’ | Not applicable  Yes  No  Not sure | | Not applicable  Yes  No  Not sure | | Not applicable  Yes  No  Not sure | | Not applicable  Yes  No  Not sure | |
| d. | **Drug interaction alerts** | Not applicable  Yes  No  Not sure | | Not applicable  Yes  No  Not sure | | Not applicable  Yes  No  Not sure | | Not applicable  Yes  No  Not sure | |
| e. | **Multi level control for prescribers**  ‘different levels of authority tailored per prescriber’ | Not applicable  Yes  No  Not sure | | Not applicable  Yes  No  Not sure | | Not applicable  Yes  No  Not sure | | Not applicable  Yes  No  Not sure | |
| f. | **Prescribing by selecting a drug from a drop down (or similar) menu** | Not applicable  Yes  No  Not sure | | Not applicable  Yes  No  Not sure | | Not applicable  Yes  No  Not sure | | Not applicable  Yes  No  Not sure | |
| g. | **Access to drug management information**  *e.g. BNF, policies, guidelines, formulary* | Not applicable  Yes  No  Not sure | | Not applicable  Yes  No  Not sure | | Not applicable  Yes  No  Not sure | | Not applicable  Yes  No  Not sure | |
| h. | **Allergy checker**  *e.g. electronic alert appears on screen* | Not applicable  Yes  No  Not sure | | Not applicable  Yes  No  Not sure | | Not applicable  Yes  No  Not sure | | Not applicable  Yes  No  Not sure | |
| i. | **Orders laboratory investigations** | Not applicable  Yes  No  Not sure | | Not applicable  Yes  No  Not sure | | Not applicable  Yes  No  Not sure | | Not applicable  Yes  No  Not sure | |
| j. | **Displays laboratory results** | Not applicable  Yes  No  Not sure | | Not applicable  Yes  No  Not sure | | Not applicable  Yes  No  Not sure | | Not applicable  Yes  No  Not sure | |
| k. | **Drug stock checking**  ‘checks if formulary drugs are available or out of stock’ | Not applicable  Yes  No  Not sure | | Not applicable  Yes  No  Not sure | | Not applicable  Yes  No  Not sure | | Not applicable  Yes  No  Not sure | |
| l. | **Discharge/transfer summaries** | Not applicable  Yes  No  Not sure | | Not applicable  Yes  No  Not sure | | Not applicable  Yes  No  Not sure | | Not applicable  Yes  No  Not sure | |

Question 27 continues on next page

| **27. Continued. Which best describes your system for each statement? Please answer all statements from (a to o) for each system you have in place.** | | | | | | | | | | | |
| --- | --- | --- | --- | --- | --- | --- | --- | --- | --- | --- | --- |
| **The system currently offers:** | | | | **System 1** | | | **System 2** | | **System 3** | | **System 4** |
| m. | | **Prompts drug administration** **by nursing staff** | | Not applicable  Yes  No  Not sure | | | Not applicable  Yes  No  Not sure | | Not applicable  Yes  No  Not sure | | Not applicable  Yes  No  Not sure |
| n. | | **Records drug administration** | | Not applicable  Yes  No  Not sure | | | Not applicable  Yes  No  Not sure | | Not applicable  Yes  No  Not sure | | Not applicable  Yes  No  Not sure |
| o. | | **If there is any other key features of the system, please specify:** | | _______________  _______________ | | | _______________  _______________ | | _______________  _______________ | | _______________  _______________ |
|  | |  | |  | | |  | |  | |  |
|  | | Please add any other comments you have. Insert the letter of the point you are referring to i.e. a. (comment) and name of system if necessary. | | | | | | | | | |
|  | | | | | | | | | | | |
| **28. Which best describes your system for each statement? Please answer all statements from (a to d) for each system you have in place.** | | | | | | | | | | | |
| **On the current system, can the following be prescribed?** | | | **System 1** | | | **System 2** | | | **System 3** | | **System 4** |
| a. | | **Continuous Intravenous infusions (IVIs)** | Not applicable  Yes  No  Not sure | | | Not applicable  Yes  No  Not sure | | | Not applicable  Yes  No  Not sure | | Not applicable  Yes  No  Not sure |
| b. | | **Sliding scale insulin** | Not applicable  Yes  No  Not sure | | | Not applicable  Yes  No  Not sure | | | Not applicable  Yes  No  Not sure | | Not applicable  Yes  No  Not sure |
| c. | | **Warfarin** | Not applicable  Yes  No  Not sure | | | Not applicable  Yes  No  Not sure | | | Not applicable  Yes  No  Not sure | | Not applicable  Yes  No  Not sure |
| d. | | **Tapering doses**  *e.g. corticosteroids* | Not applicable  Yes  No  Not sure | | | Not applicable  Yes  No  Not sure | | | Not applicable  Yes  No  Not sure | | Not applicable  Yes  No  Not sure |
|  | | Please add any other comments you have. Insert the letter of the point you are referring to i.e. a. (comment) and name of system if necessary. | | | | | | | | | |
| **29. Which drugs (if any) are prescribed on a supplementary paper drug chart?** (Please select all that apply) | | | | | | | | | | | |
|  | **System 1** | | | | **System 2** | | | **System 3** | | **System 4** | |
|  | None  Continuous IVIs  Insulin  Warfarin  Tapering doses  Other (please specify)  ________________ | | | | None  Continuous IVIs  Insulin  Warfarin  Tapering doses  Other (please specify)  ________________ | | | None  Continuous IVIs  Insulin  Warfarin  Tapering doses  Other (please specify)  ________________ | | None  Continuous IVIs  Insulin  Warfarin  Tapering doses  Other (please specify)  ________________ | |
|  | | Please add any other comments you have. Insert the name of the system if necessary. | | | | | | | | | |

| **30. Does your hospital intend to introduce a new prescribing system(s)? If Yes, when:** | | | | | | |
| --- | --- | --- | --- | --- | --- | --- |
|  | | No | <1 year | 1-2 years | >2 year | Not sure |
|  | Please add any other comments you have. | | | | | |

| **31. Please provide your details below if you are happy for us to contact you in case any of your responses require further clarification. Your contact information will remain confidential.** |
| --- |
| Name: __________________________________________    Role/job title: ___________________________________  Email address: ___________________________________  Phone number: ___________________________________  Bleep number: ___________________________________ |

| **32. Thank you for completing this survey. The information you have provided will help us to identify what medication systems are currently in use and will contribute to the development of future strategies to (1) reduce medication errors, (2) streamline hospital medication systems and (3) reduce wasted medications. Would you be willing to be contacted for the next stage of our research?** |
| --- |
| Yes  No |
